# Non-Invasive Manipulation of Two-Phase Liquid–Liquid Slug Flow Parameters Using Magnetofluidics

**DOI:** 10.3390/mi12121449

**Published:** 2021-11-26

**Authors:** Anoj Winston Gladius, Simon Höving, Mehdy Mendelawi, Harikrishna Sreekumar Sheeba, David W. Agar

**Affiliations:** Laboratory for Chemical Reaction Engineering, Department of Biochemical and Chemical Engineering, TU Dortmund University, Emil-Figge-Straße 66, 44227 Dortmund, Germany; simon.hoeving@tu-dortmund.de (S.H.); mehdy.men@gmail.com (M.M.); harikrishnaas93@gmail.com (H.S.S.); david.agar@tu-dortmund.de (D.W.A.)

**Keywords:** liquid–liquid slug flow, magnetofluidics, micropumps, micromachines, microcapillaries

## Abstract

Liquid–liquid slug flow in a microcapillary, with its improved heat and mass transfer properties and narrow residence time, plays a vital role in process intensification. Knowledge of the flow properties in microchannels along variables’ controllability (e.g., phase ratio, slug length along with classical variables, such as pressure, temperature, and flow velocity) during operation is crucial. This work aids in this by using magnetofluidics to manipulate these parameters. A ferrofluid with reproducible properties is produced and, together with another phase, stable slug flow is generated. Micro-gear pumps and syringe pumps, with their traditional mechanical components, result in parts degrading over time due to fatigue caused by pressure differentials and corrosive chemicals. The microflow is also disturbed by the invasive nature of these pumps. A considerably energy-efficient, non-invasive alternative, with reduced mechanical interfacing is suggested in this work. It uses magnetic gradients to manipulate two-phase flow, one of which is a magnetically active phase. Conveying concepts using permanent magnets in the immediate vicinity of the flow are investigated. To operate this pump continuously and to be able to regulate the phase ratio, an electromagnetic non-invasive valve is developed. Phase separation is also carried out with an existing decanter design, modified using electromagnetism to work without a selective membrane, usually necessary for phase separation at this scale. This pump is then compared with similar pumps developed in the past.

## 1. Introduction

To produce fine and special chemicals, simple and safe handling and control of processes are essential. With small component dimensions and small throughputs, micro-process engineering offers interesting possibilities for implementing complex processes [1,2,3,4,5]. Operations in this field have great potential in terms of achievable performance in heat and mass transfer properties [2,3,5]. In multiphase flows, dispersions with very favourable volumetric surface ratios are achieved and processes limited due to mass transfer become attractive again on a smaller scale [6]. Due to the intensive mixing of liquid and gaseous reactants, the achievable transfer rates and turnovers exceed conventional process technology by orders of magnitudes [7,8]. Furthermore, higher selectivity, yield and product quality follow from the narrower range of residence time distributions [8]. Due to the short development and product introduction times of micro-processes, their use is particularly important in the pharmaceutical industry [9]. Here, the products are usually protected by patents with a term of 20 years and shorter time-to-market periods enable profits to be maximised [9]. Numbering up of these microchannels in parallel would result in reaching industrial output [10,11]. 

To optimally utilise the advantages of micro-process engineering, along with precise knowledge of the flow properties and the processes in the microchannels, various process variables need to be changed during operation to adapt them to different conditions [10,11,12]. This work deals with the non-invasive manipulation of two-phase liquid–liquid slug flow parameters. 

The liquid–liquid slug flow is a two-phase flow regime that occurs in capillaries, offering several characteristics beneficial to the performance and controllability of two-phase reaction systems. It is made up of two immiscible liquids where one liquid shows a higher affinity towards the capillary material. The high-affinity liquid forms a continuous phase in which the other liquid is present in the form of dispersed slugs, separated from the capillary by a thin wall film.

In the case of two-phase flows in microchannels, the phase ratio and the slug length are added to the classic process variables in a chemical reaction system such as pressure, temperature, or flow velocity. Adjusting slug length and phase ratio can be of great importance for various processes in micro-process engineering to create ideal reaction conditions [12]. Gladius et al. deal with an example of such reaction systems where slug flow parameters are used to control selectivity towards products in an oxidation reaction [3].

Previous approaches to varying the phase ratio and slug lengths at arbitrary flow rates aim, for example, at adjusting the mixing chamber volume by an invasive intervention. This is the case as Arsejuk et al. describe a slug generator setup, one amongst many such existing invasive designs to manipulate two-phase liquid–liquid slug flow systems [13]. This device guides the continuous and disperse phase via a coaxial channel into a conical mixing chamber. The central channel applies the disperse phase via a height-adjustable needle into the mixing chamber. By lowering or raising the needle channel, the volume of the mixing chamber is varied, successfully resulting in shorter or longer slugs [13]. However, this invasive method of slug generation has disadvantages. The height adjustment of the needle causes pressure fluctuations, which disrupts the inflow of the continuous and disperse phases. In addition, the formation of undesirable side slugs is also observed in reaction studies done by Gladius et al. [3]. Other disadvantages include the use of moving, degradable components and the need to seal them from the environment during translational and rotational movements of the needle. Sealing failure can be observed at high operating pressures, as seen in the case of Gladius et al. [3], where high operating pressures result in fluctuations in the flow conditions due to the control systems employed to regulate the mechanical components. A combination of high operating pressures and hazardous materials is therefore not recommended. In addition to the needle position, the final slug length depends on other influencing factors, such as the flow rates of the individual phases, adhesive properties of the construction material and the geometry of the slug generator, which cannot be actively changed. The height adjustment of the needle is an active process, while the actual slug formation is a passive process [10]. Accordingly, an active shaping mechanism in slug formation is a challenge, as can be seen in the control system design complication involved in the studies carried about by Arsenjuk et al. 

The change in the flow can also be made using mechanical internals such as needle valves [4]. At these microscales, the slug flow properties are interlinked with each other and manipulating one results in another being affected, as explained by Arsenjuk et al. in their work [10]. This poses a challenge especially at high process pressures or very unstable chemical systems [10]. So, any kind of non-invasive, non-mechanical intervention is sought after, hence justifying the search for further possibilities to independently vary the characteristics of a liquid–liquid flow in microchannels.

Non-contact force transmission and manipulation of rheological behaviour offer many possibilities for micro-process engineering operations. In the field of non-contact manipulation and measurement, ideas including thermo-rheological valves, dielectric measurements, etc., have been successfully tested [10]. Besides the disadvantage of high-pressure loss across the reservoirs [10,13], the control of a large number of parallelized microcapillaries using non-invasive thermo-rheological valves is particularly challenging with parallelized multiphase reaction systems [10]. The necessity for the existence of a complicated control system is guaranteed, as each microcapillary must have the same flow and, thereby, non-fluctuating reaction conditions. This is either achieved using a separate pump and sensor system for each strand or using central conveying units paired with liquid distributing units. However, this method of control is not economically feasible for a large number of parallelized microcapillaries as manufacturing tolerances in these micro-capillaries might lead to the maldistribution of fluids.

To facilitate easy, cost-effective, non-invasive manipulation in liquid–liquid slug flow, the potential of magnetofluids is investigated in this work. Magnetofluids are suspensions consisting of magnetic particles and a carrier liquid. Magnetic particles can be maghemite, magnetite or cobalt-iron alloys. Water or various oils are typically used as carrier fluids [14]. An interesting property of magnetofluids is that in the presence of a magnetic field, they can drastically change various properties. Among the most important effects is the change in viscosity, which is used, for example, in seismic dampers [15] and dynamic sealing systems [16]. The attractive forces of magnets on magnetofluids can be exploited to slow down, accelerate or hold magnetofluids in closed channels [17]. Magnetofluids with different properties are distinguished into magnetorheological fluids and ferrofluids [18]. In magnetorheological fluids, particles are present on a micrometre scale, suspended in a carrier fluid resulting in typical properties of magnetofluidics, however, the particles are subjected to a high degree of classical sedimentation effects induced by magnetic fields. Depending on the application, this effect, along with frequently occurring agglomeration amongst larger particles, can lead to a loss of the desired properties [19]. Ferrofluids, on the other hand, are characterised by colloidally distributed ferromagnetic nanoparticles of single domain nature. The magnetically active particles in these fluids are so small that they are not subject to sedimentation effects, allowing them to remain suspended due to Brownian motion. Ferrofluids can therefore be used for applications where a continuous and homogeneous phase is mandatory [20]. Kurtoğlu et al. clearly showed that a homogenous system of magnetically active nanoparticles can be used for this purpose [21]. The synthesis and further details regarding the ferrofluid produced and used in this work are further explained in Appendix A. 

Pumps and conveying technologies are becoming increasingly important in micro-process engineering [22]. In addition to the classical applications in pharmacy for drug dosing [23], micropumps are increasingly used in other areas, such as fuel dosing for micro-fuel cells [24] or biochemical applications [25]. There has also been extensive progress in the field of non-invasive delivery of ferrofluids [21,26,27,28]. This method of transport is mainly used for two-phase flows in microcapillaries. In these flows, the phases should alternate evenly, with the magnetically active phase being discontinuous. When the magnetic phase is attracted by an induced magnetic gradient, the moving slug creates a pressure differential that is ideally large enough to set the entire flow in motion [14]. 

In addition, a distinction is made here as to whether the gradient is created by mechanically moving permanent magnets or by the pulsation of electromagnets. As shown by Kurtoğlu et al., a two-phase flow can be induced in a microcapillary by moving permanent magnets through rotation or conveyor belts [21]. By changing the position of the magnets with respect to the ferrofluid, a magnetic field gradient can be created which is strong enough to pull the fluid through the capillary. Joung et al. have presented a method in which mutually offset tips are alternately magnetically activated to deliver microparticulate iron through a fluid [29]. The magnetised tips are all of the same polarity. All tips correspond to the magnetic north pole. 

The promotion of two-phase flows by exploiting magnetic activity has already been described several times [21,29,30,31]. Although all investigations differ in details, such as the continuous phase, the type and production of the nanoparticles, the carrier phase or the production method of the actual pump body, some characteristics that are similar for all investigations can be singled out: all concepts are based on the fact that the carrier fluid of the nanoparticles in the two-phase mixture in the microchannels results in the discontinuous phase. While the carrier fluid takes on a black colour due to the nanoparticles, the continuous phase remains transparent. On the one hand, this simplifies the use of inexpensive optical sensors and, on the other, provides information about the stability of the ferrofluid, since extraction processes can be easily observed through discolouration of the continuous phase [21].

**Figure 1:**
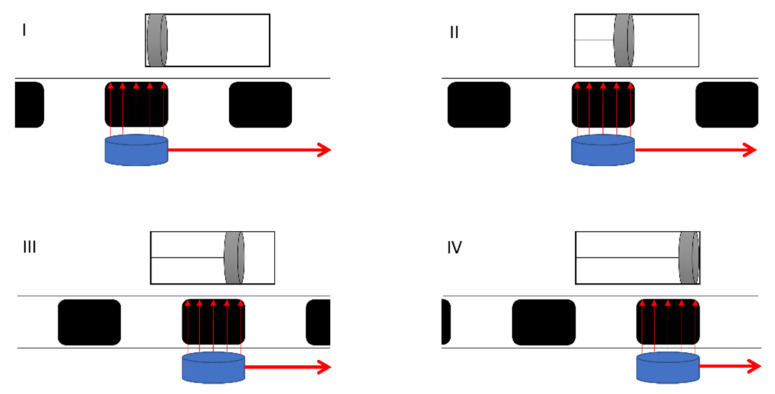
Analogy between slug flow and a piston pump. Each slug corresponds to a new piston. (**I**) Ferrofluid starts to accelerate; (**II**) Ferrofluid reached maximum acceleration; (**III**) Ferrofluid starts to decelerate; (**IV**) Ferrofluid stops accelerating but moves due to existing inertia.

However, it is important to distinguish whether the investigations described are rudimentary movements or actual pumping of the flow across a pressure gradient. In the case of rudimentary pumping, the ferrofluid is merely drawn through the continuous phase by an external magnetic gradient. This pumping has been achieved, for example, by Joung et al., slugs breaking off is a big problem. During actual pumping, the ferrofluid slugs remain intact and are pulled through the channels without breaking up. This creates a pressure differential that ideally sets the continuous phase and thus the entire flow in motion. All investigations in which actual pumping could be achieved have this basic principle in common. The piston pump serves as an analogy to technical pumps. By moving a ferrofluid slug, a low-pressure region is created, similar to a piston pump. This draws in fluid and sets the flow in motion. The sequence of the pump steps is shown in simplified form in Figure 1. In this simplification, the required magnetic field gradient is generated by a movable magnet that is guided along outside the microchannel. The slugs are moved in such a way that, in contrast to the classic piston pump, no valves are required, since the delivery piston renews itself with each new slug. In continuous delivery, steps I to IV are run through periodically.

This work has tried to establish such a pumping concept along with a concept to magnetically alter the phase ratio and slug length independently and a concept to magnetically decant the magnetic particles from the slug flow, thus facilitating regeneration and recycling of the magnetofluids. This system can be directly used for reaction systems that require iron components as catalysts. Other reaction systems could be potentially modified to work with this concept as well.

## 2. Magnetic Propulsion

The propulsion of a two-phase flow by exploiting the interaction between an electronically induced magnetic field and ferrofluid was not very successful. The main reason for this is the strong local fluctuations in the magnetic fields, which caused the deformation and rupture of the slugs. However, during the investigations, it became apparent that permanent magnets can be used to pull the ferrofluid slugs through the capillary. For this purpose, neodymium magnets are guided parallel to the capillary. It can be observed that the slugs deform in the capillary, but only tend to tear apart when they reach a certain size. In addition, it can be observed that especially with long slugs and short capillaries, the positioning of a single slug sets the entire flow in motion. 

**Figure 2:**
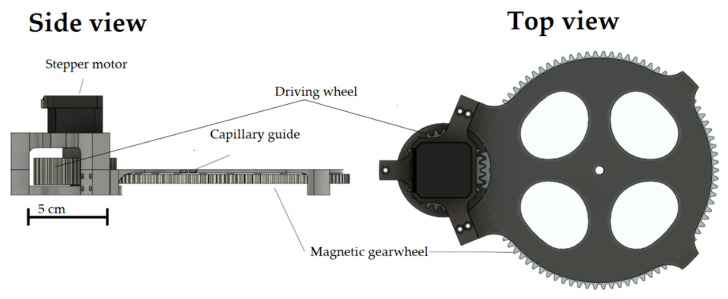
CAD drawing of the pump structure in side view (**left**) and top view (**right**).

Cylindrical neodymium magnets (Supermagnete, Gottdamingen, Germany) with a diameter of 6 mm and a height of 3 mm are used as permanent magnets for all investigations. The magnets are embedded in a 3D-printed gear wheel as shown in Figure 2. The gear wheel rotates above the base of the assembly, which is also printed, in which the capillary is embedded in a circular path. The inner diameter of this circular path corresponds to 2 mm so that a PTFE capillary with an inner diameter of 1 mm and an outer diameter of 1.6 mm can pass through. The diameter of the circular capillary guide and the diameter of the circular track on which the neodymium magnets are mounted is 164 mm. An M8 thread is printed at the centre of the base to fix a threaded rod in it as a centring aid. A ball bearing with an inner diameter of 8 mm is embedded in the centre of the magnetic gear wheel to enable centring using the threaded rod fixed in the base. The magnetic gear is driven by another gear with a smaller number of teeth, again driven by a Nema 17 stepper motor. The transmission ratio of the drive gear to the magnetic gear is 3:1. To enable more precise control of the rotation speed at low speeds, the stepper motor is driven by a TMC2208 driver (Renkforce, Hirschau, Germany). The stepper motor is powered by the driver using a 12-V power supply (Hewlett-Packard, Palo Alto, CA, USA). The driver is controlled by an Arduino Nano.

**Figure 3:**
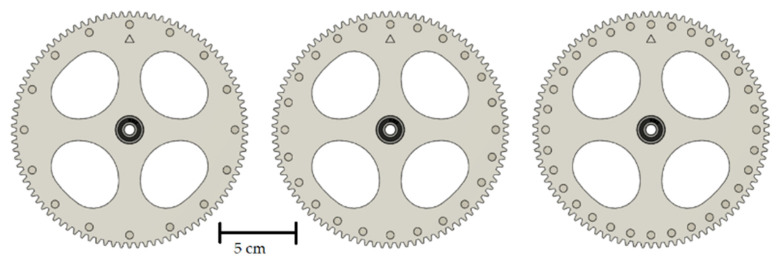
Gears with 16, 24 and 32 magnets (from top to bottom).

**Figure 4:**
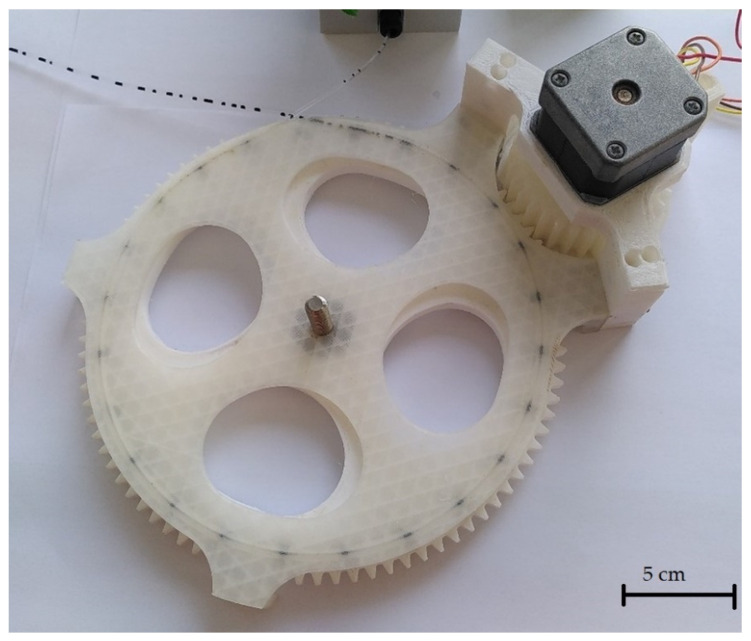
Design of the magnetic gear pump with recessed capillary and an uneven two-phase flow.

To create different slug lengths, the magnetic gear wheel must be replaced in the design shown. Therefore, a total of three different versions of the gear wheel are designed and manufactured. The different gears have the same number of teeth and the same diameter but a different number of magnets. The designs of the gears are shown in Figure 3. To continuously supply the flow with liquid, a two-phase flow must already be present in the capillary when it enters the capillary holder. This two-phase flow is generated by a simple T-mixer. The entire assembly consists of a total of five 3D-printed components, of which the three immovable parts of the motor bracket are fastened together using M3 screws and nuts. The pump described is shown assembled in Figure 4.

To pump fluid in the capillary, the maximum number of magnets must continuously interact strongly with the ferrofluid. The circular arrangement of the magnets and the capillary is intended to ensure that almost all magnets interact with a slug at any given time and the design using two plates ensures that all magnets are pressed onto the holder with uniform force, thus keeping the distance constant on the one hand and minimal on the other. A stepper motor is chosen because it can be driven with less fluctuation in the speed of rotation compared to a classic DC motor. Several factors speak in favour of a drive using gear transmission and against a direct drive of the disc with the embedded magnets. Firstly, the equipment required to place the stepper motor in the centre of the magnetic disk is significantly higher than to position it at the edge. In addition, stepper motors, even with micro-steps, do not work completely pulsation-free. Employing the 3:1 ratio, the pulsation effect of the rotation is reduced and a more even conveying is made possible. 

With a constant phase ratio, the slug distances are influenced by the distance between the magnets and cannot be changed during operation. To nevertheless produce and also convey slugs of different lengths, a total of three different magnet discs are manufactured. First, it is investigated whether a basic functionality of the described pump can be shown. For this purpose, a two-phase mixture of ferrofluid and kerosene is introduced into the pump from the T-mixer. In addition to the delivery, the slug coalescence in the pump is also to be investigated.

It is proved that with the described setup, a continuous conveyance of a two-phase flow is possible. The magnets in the gear wheel interact so strongly with the ferrofluid slugs that these are also set in motion by a movement of the gear wheel. Here, the attraction is strong and even, avoiding the deformation of the slugs. The moving slugs, therefore, create the pump effect. Due to the large number of slugs moving at the same time, the pressure differential created is strong enough to attract ferrofluid and kerosene through the T-mixer from the receiver tanks and to convey further slugs into the pump as a two-phase flow, without the aid of any mechanical pumping devices. 

**Figure 5:**
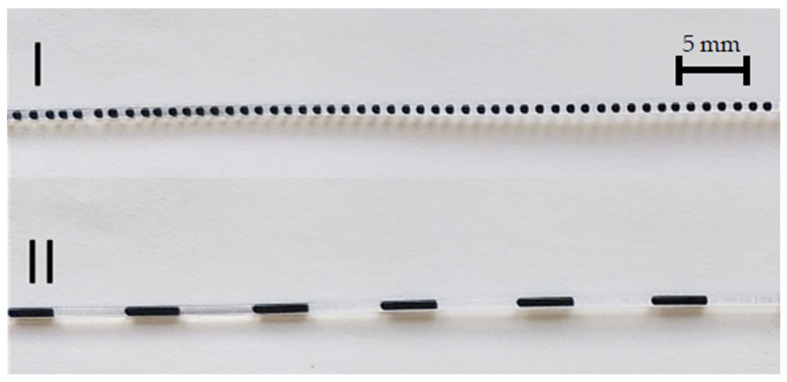
Slug flow in the capillary before (**I**) and after (**II**) the pump.

In addition to the actual conveyance of the flow, slug coalescence can also be observed in the pump. The length and spacing of the slugs before entering the pump are determined by the flow characteristics in the T-mixer. With the flows and characteristics present, a two-phase flow is produced as shown in Figure 5I. The distances between the slugs are smaller than the distances between the magnets in the magnetic gear. Therefore, each magnet attracts nine to ten slugs, which then combine to form a larger slug. The average distance between the slugs is given by the distances between the magnets on the gearwheels. The actual distance between the slugs also depends on the phase ratio. The flow generated by the pump is shown in Figure 5II.

**Figure 6:**
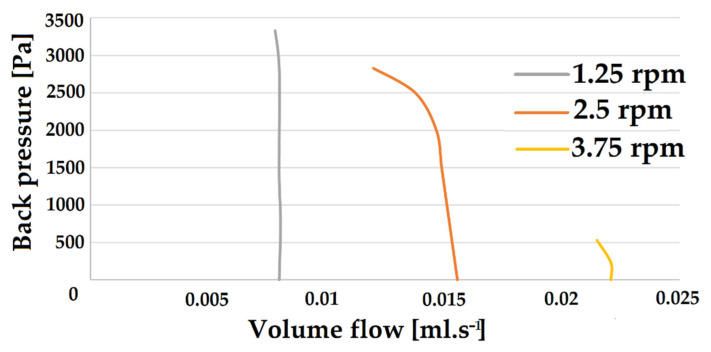
Pump curves for three different operating conditions.

To describe the pumping characteristics of the described pump in more detail, pump curves for the magnet gear with 24 magnets at a constant phase ratio are first investigated and quantified. For this purpose, the pump is operated at three different speeds and both the pump pressure, and the volume flow are determined to record a total of three pump characteristics. The pump curves determined for the three speeds of rotation of the magnetic gearwheel are shown in Figure 6. For a low speed of the magnetic gear, a pump characteristic can be seen, which is typical for piston pumps. The line is almost perpendicular to the X-axis and only bends slightly at high heads. At higher speeds, however, a kink in the pump curve can be seen much earlier. The characteristic of these curves can be seen very well at a speed of 2.50 rpm. At low back pressures, the pump characteristic curve resembles the typical vertical characteristic curve of a piston pump. 

At higher back pressures, however, a rounding of the characteristic curve can be seen, as can be observed with centrifugal pumps. This mixed form of the pump characteristic curve can be explained by the fact that the pump described has characteristics of both pump types. On the one hand, the pumping of the fluid is made possible by the movement of the slugs. On the other hand, however, the magnetic gear wheel above the capillary rotates, similar to the feed wheel in a centrifugal pump. Particularly with high back pressures, a kind of slippage can occur. The magnets then continue to rotate at a constant speed, but the interaction with the ferrofluid slugs is not strong enough to deliver the entire flow at the same speed. Individual parts of the slugs break off and unite with the following slug. The actual flow speed then decreases despite the constant rotation speed of the magnet.

In microfluidics, the associated pressure loss is of interest due to the small diameters and wall friction effects. Using the determined pump characteristics, the maximum pressure losses that can be overcome can be determined. To assess the possible applications, the maximum delivery length of a pump for a specific capillary or channel may also be of interest. Here, the capillary used has an internal diameter of 1 mm. 

Equation (A1) (Appendix C) is used to determine the maximum capillary lengths for the operating conditions shown in Figure 6. For density and viscosity, the values of the continuous phase, i.e., the kerosene, are used. The determined maximum pressures, volume flows at maximum pressure and the maximum capillary length are listed in Table 1.

It can be observed that at lower rotation speeds, lower volume flows but higher maximum pressures can be achieved. The highest pressure is reached at a rotational speed of 1.25 rpm. At a flow rate of 0.0078 µL s^−1^, the pump works against a pressure head of 10.47 m through the described capillary. At higher flow rates, only significantly lower pressures can be overcome, but especially for processes in microfluidics, this may be sufficient depending on the application. To compare the three manufactured magnetic gears with each other, the maximum volume flow at the minimum head is determined for each gear. The maximum achievable volume flows for the three different magnetic gears are shown in Table 2.

For all gears, it is observed that when the rotation speed is increased, the achievable flow rate increases approximately linearly. This effect can be seen in the pump curves in Figure 6. However, after a certain point, slippage effects become more and more pronounced, and a further increase of the rotation speed leads to a standstill of the flow. The number of magnets is used to influence the maximum achievable volume flow. The more magnets are installed in the gears, the shorter the slugs in the capillary become. This in turn causes the ferrofluid to be closer to a magnet on average and thus interacts more strongly with them. Due to the stronger interaction, higher speeds are then possible without the slip effects prevailing. The maximum volume flow is achieved at a rotational speed of 11.28 rpm with a gear wheel with 32 magnets and is 100 µL s^−1^.

To be able to compare the pump with existing systems, the power consumption is determined at a volume flow of 30 µL s^−1^. In the pump described, a large part of the consumed energy is used to drive the stepper motor. This is supplied with a TMC driver, which can be driven with operating voltages of 12 V to 35 V. The driver also has an internal voltage converter that delivers a constant voltage of 5 V necessary to supply the Arduino microcontroller used for control. If a flow with a volumetric flow of 30 µL s^−1^ is conveyed, a current of 0.16 A is measured at a voltage of 15 V. This results in a power of 2.4 W. To classify this value, a two-phase flow with the same phase ratio and flow velocity is generated using syringe pumps of the type, LEGATO 100 (KD Scientific Inc., Holliston, MA, USA). 

To generate a comparable two-phase flow, two of these pumps are needed, which then generate the slug flow using a T-mixer. In the syringe pumps described, a current of 0.3 A is measured at a voltage of 12 V when generating this flow. Both pumps together consume about 7.2 W and, thus, about three times as much as the magnetic gear pump. Although these power consumptions are only comparable to a limited extent, since the syringe pumps are powered by complex electronics and a display in addition to the motor, they show that the developed pump can be more economical than conventional syringe pumps.

To increase the delivery volume flow and the maximum achievable pressure, some optimisation is attempted. To intensify the magnetisation of the slugs, another disc with the same number and the same orientation of magnets is added to the setup shown in Figure 4. Two different approaches are being investigated here. First, the magnets in the second magnetic gear are inserted in such a way that the magnets face each other with opposite poles. In other words, the gears attract each other. The manufacturer states that the neodymium magnets have a holding force of 4 N at 1.6 mm when two magnets with opposite poles attract each other. With a total of 24 pairs of magnets, this corresponds to a force of 96 N with which the two gear wheels are attracted to the capillary holder. 

The capillary holder and the capillary itself are not damaged due to the evenly distributed force. However, the holding force is too great to be overcome by the stepper motor and can no longer be moved in this orientation. An improvement or deterioration of the pump characteristics is hence not observed. In addition, a same-pole alignment of the magnets against each other is investigated, where it is assumed that the two magnet gears are pressed apart with a force of 96 N in total. 

However, this force is too high for the comparatively high tolerances in additive manufacturing methods and the material used (PLA) compared to traditional methods. The gears and the holder are pressed apart so strongly that individual teeth of the gears are skipped. As a result, the magnets are no longer placed directly above each other. Conveying with magnets placed in opposite poles can therefore also not be assessed with the setup described.

## 3. Phase Ratio Setting Using Magnetofluidics

**Figure 7:**
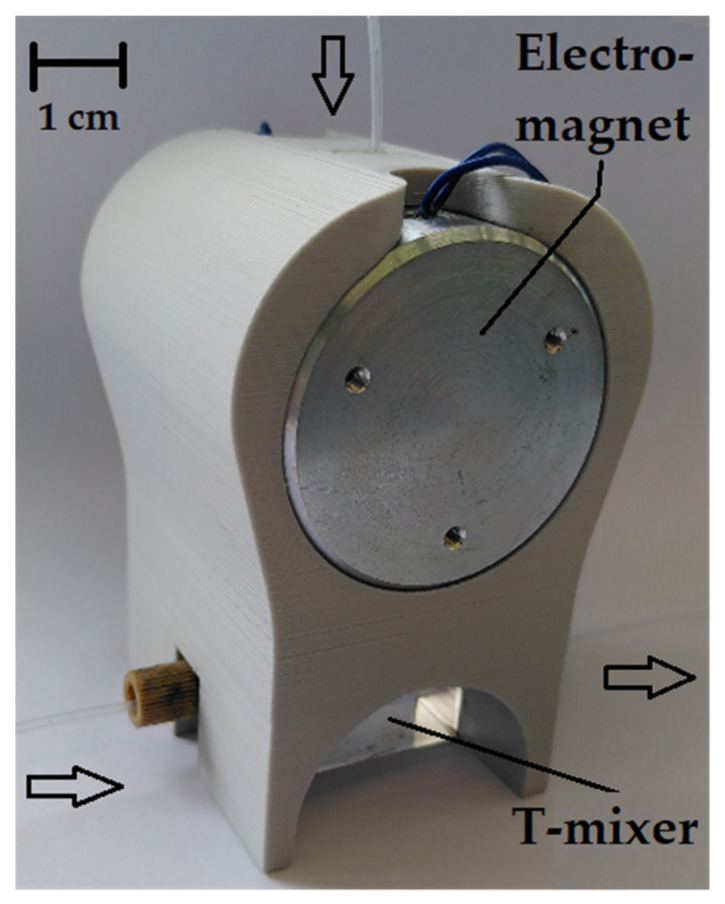
3D-printed setup of the phase controller with connected capillaries.

In addition to pumping the flow and changing the slug length, one of the goals of this work is to adjust and regulate the phase ratio of the components of the flow. A uniform phase ratio is also important to enable the continuous operation of the pump. For this purpose, the characteristic property of the ferrofluid is used in that the viscosity is increased under the influence of a magnetic field. As described in the previous sections, a simple T-mixer is used to mix the phases. The continuous phase, the kerosene, is fed into one inlet of the mixer and the discontinuous phase, the ferrofluid, is fed in from above. A holder is placed on the T-mixer into which two electromagnets, each with a diameter of 50 mm, can be inserted. Behind the output of the T-mixer is an infrared photoelectric sensor of the type TCUT 1300X01 which determines the phase ratio employing an Arduino. This technology has already been used by Arsenjuk et al. [10]. The phase ratio is used as a control variable for a PI controller that is integrated with the Arduino and regulates the phase ratio. The voltage with which the electromagnets are supplied serves as the control variable. The voltage is provided by a high current source, which in turn is controlled by the Arduino. The Phase Ratio controller is shown in Figure 7. 

The T-mixer with an attached, non-invasive magnetic valve is connected in front of the pump described in the previous section. The pressure differential generated by the pump enables continuous delivery. To check the functionality of the phase controller, a disturbance is introduced during continuous operation. This is done by raising the feed tank of the ferrofluid by a few centimetres for a short time. This increases the pre-pressure of this phase and the phase ratio in the capillary changes. Controlling the phase ratio is only possible if a pressure gradient that is as continuous as possible draws both fluids into the T-mixer. The pumps on the other side can only deliver continuously if the phase ratio is as uniform as possible.

**Figure 8:**
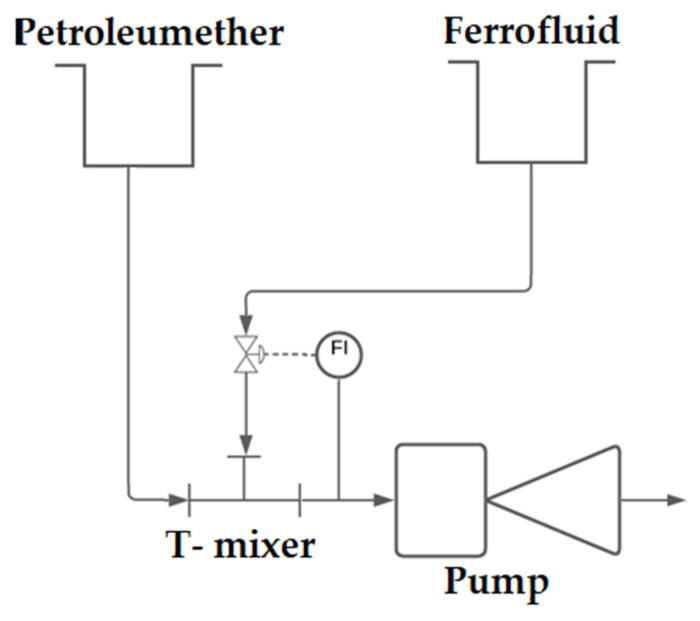
Continuity validity setup for the Phase controller—magnetic pump setup.

The validation of the continuity of both variables is therefore determined together, as shown in the experimental setup in Figure 8. Both concepts are tested together by examining a total of nine combinations of phase ratios and average slug distances. The phase ratios are set to 0.25, 0.5 and 0.75 by programming the Arduino. The mean slug distances are set to 14.5 mm, 21.6 mm and 28.6 mm by the manufactured magnetic gears. To change the diameter of the capillary or the magnets from maximum to minimum, the two discs that belong together must be operated with a phase difference of 45°. 

Although the initial experiments have proven the basic functionality of the described controller, a weakness is revealed and it becomes apparent when the proportion of ferrofluid is greater than the setpoint, and the electromagnet is deactivated. In this case, the proportion is not further reduced by further decreasing the voltage. However, since the pump system described only applies a few millibars of suction pressure, this deficit can be compensated or reduced by increasing the liquid level in the ferrofluid reservoir. The higher liquid level of the ferrofluid compared to the kerosene ensures a larger proportion of the ferrofluid in the outgoing flow in the case of uncontrolled mixing in the T-mixer. Reducing this proportion is more feasible with the design described than increasing it. Although a more stable and reliable control solution is to be found in the medium term, this control is sufficient to test the functionality of the phase ratio control and the pump.

**Figure 9:**
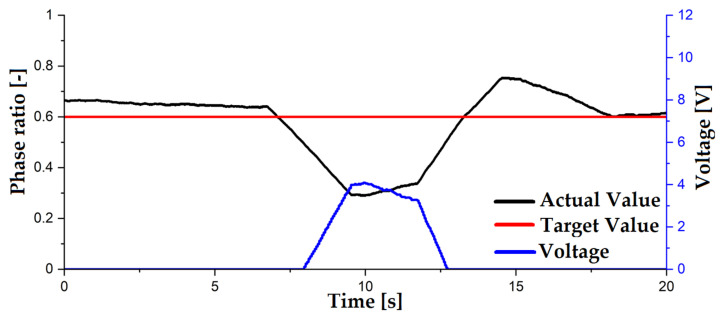
Dynamic actions of the actual and setpoint value of the phase ratio and voltage.

Due to the low delivery pressure and the dependence of the phase ratios on the hydrostatic pressure, a simple lifting of one of the receiver tanks introduces a disturbance into the system. Lifting the ferrofluid receiver increases the hydrostatic pressure of this phase. This increases the phase ratio in the capillary. This introduced disturbance and the resulting time courses of the actual and setpoint values of the phase ratio and the course of the voltage are shown in Figure 9. In red, in Figure 9, the set value of the phase ratio is shown, in black, the actual value and, in blue, the voltage with which the electromagnet is supplied in volts. A phase ratio of zero means a capillary filled with ferrofluid and one means a capillary filled with kerosene. After about six seconds, a disturbance is introduced by raising the ferrofluid reservoir a few centimetres. This increases the hydrostatic pressure for this phase and the proportion increases. The actual value drops to about 0.3. After the disturbance is introduced, the controller reacts and increases the voltage of the electromagnet. This creates a strong magnetic field in the immediate vicinity of the feed capillary, which increases the viscosity of the fluid. As a result, the proportion of ferrofluid in the capillary decreases again and the phase ratio increases. After the actual value briefly exceeds the set value, it stabilises again at the pre-set 0.6. It is thus shown that the phase ratio in the microcapillary can be controlled using an electromagnet.

**Figure 10:**
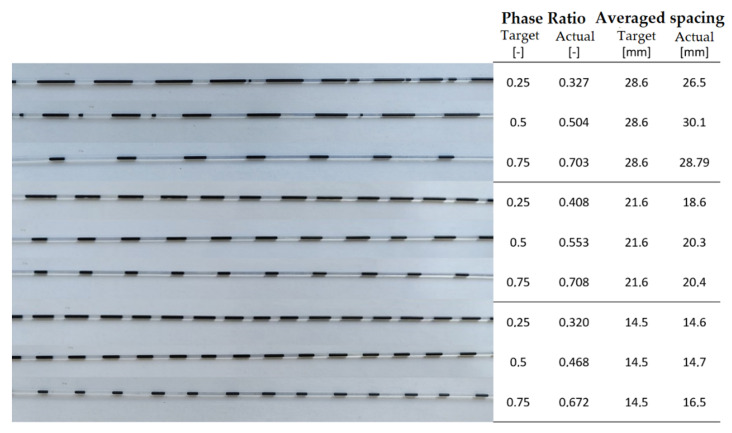
Flow conditions at different phase ratios with values of the phase ratios and the averaged distances.

Since the mean slug spacing (for more details on the term, refer to Appendix B) can now be changed using the exchangeable magnetic gears and the phase ratio can be changed using the control described above, the continuity of both variables is considered simultaneously. The results of the experiment described above are shown in Figure 10. The distances between the slugs are determined by the magnet gears, the phase ratios can be adjusted by setting the PI controller. Due to the geometry of the gears, the distances of the magnets on the circular path are set as 28.6 mm, 21.6 mm, and 14.5 mm. The phase ratios are set as 0.25, 0.5 and 0.75. When setting the phase ratios, the first thing to notice is that deviations from the programmed phase ratio fluctuate slightly towards the centre. If the set value is 0.75, the actual value is below this in all three states; if the set value is 0.25, the actual value is slightly above this in all states. Phase ratios of 0.5 can be set for all three gears with very little deviation.

The average slug spacing (for more details on the term, refer to Appendix B) can be adjusted well for all states and deviates only slightly from the set values. However, increased slug breakage can be observed especially with large distances and a high proportion of ferrofluid. Since the slugs become longer overall with a high proportion of ferrofluid and large average distances, the amount of ferrofluid attracted by each neodymium magnet also increases. However, if the slugs become too long, the strength of the magnets is no longer sufficient to pull them through the capillary as a whole. At the end of the slugs, smaller slugs break off, which are then no longer attracted by any magnets and are only transported through the capillary by the pressure differential. 

It is then shown that the phase ratios and the average slug distances can be reliably adjusted using the described pump and valve. During operation, the phase ratios of the pumped flow can already be adjusted. The average distances between the slugs are still determined by the gear wheels and the magnets embedded in them and can therefore not be changed during operation. An extension of this design that allows the average slug spacing to be changed is described in Section 6.

## 4. Phase Separation and Continuous Plant Operation

Now that a functioning continuous generation and delivery of a two-phase flow has been implemented, the next step is to consider the separation of the two-phase flow. The water (used as the carrier fluid for the ferrofluid) and the continuous phase of the kerosene are immiscible and can therefore be separated from each other by decanting. The magnetic activity of the ferrofluid will further accelerate the separation caused by the density difference. 

To enable a continuous separation of the two phases, a selective membrane is classically used [32]. This membrane enables continuous separation even with uneven phase ratios. Suitable sensor technology enables operation without a selective membrane. In this way, the pressure losses that occur due to the membrane can be bypassed. An already existing and used decanter designed by Schwarz et al. is modified so that it can be used for the separation of the described flow [32]. The decanter consists of various cylindrical acrylic glass modules which are provided with holes and screwed together using a total of 6 M5 threaded rods. The individual modules have a diameter of 65 mm and a height of 10 mm or 15 mm. 

**Figure 11:**
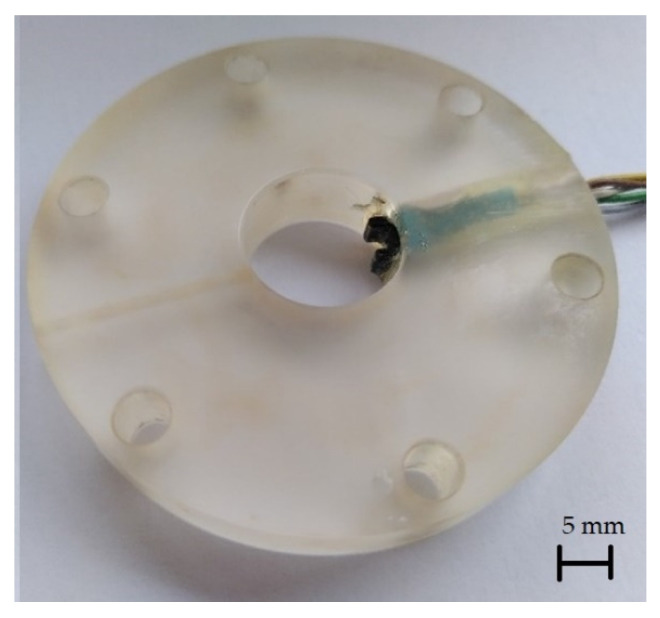
Individual disc module of the decanter with glued-in TCUT optical sensor for level control.

In two of these modules, the existing holes are enlarged so that optical sensors of the TCUT type can be glued into the holes. Employing transparent two-component adhesives, the optical sensors are not only fixed in the modules but also the borehole with the cables passing through it is sealed tight. It is important to ensure that a thin but continuous layer of adhesive of about 0.1 mm is distributed over the entire sensor surface. This layer does not restrict the function of the optical sensor, but it does prevent the sensor from being impaired by interactions with the ferrofluid or kerosene. The module described is shown in Figure 11. 

**Figure 12:**
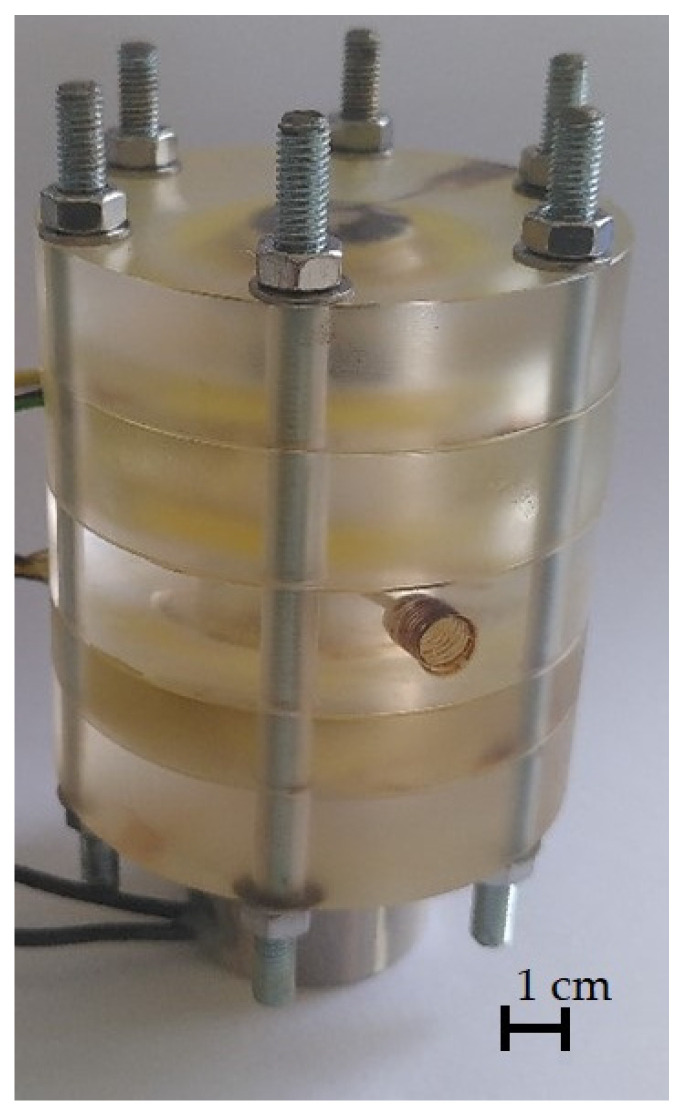
Assembled decanter consisting of a total of 5 acrylic glass modules.

A total of two of the modules shown are manufactured with built-in sensors. The modified modules are assembled with a total of three other modules in such a way that a liquid-tight interior with a total of three connection options for microcapillaries is created with a fitting. The modules are sealed with PTFE sealing tape and firmly screwed together using M5 threaded rods. The lowest module of the decanter stands on an electromagnet, which is connected to a voltage source via a relay. The entire construction is shown in Figure 12.

**Figure 13:**
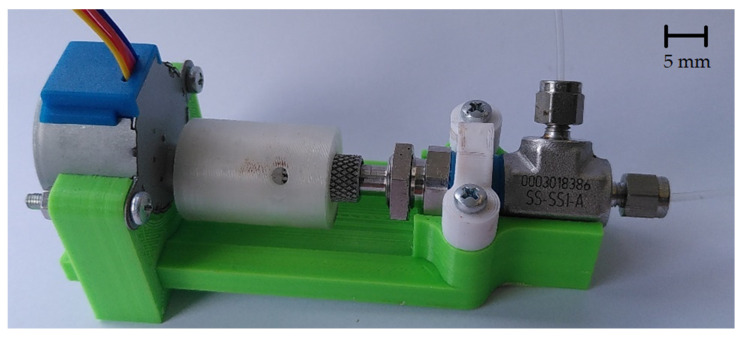
Needle valve with 3D-printed modification to allow operation using a stepper motor.

The two-phase flow enters via the middle section of the decanter. The light phase is discharged through the upper port and the heavy phase through the lower port. A needle valve (Swagelok, Solon, OH, USA) as shown in Figure 13 is attached to each outlet. The opening state of the needle valves is changed by a 28BYJ-48-5V (Makerfactory, Hirschau, Germany) stepper motor. An Arduino is used to evaluate the signals of the optical sensor (Makerfactory, Hirschau, Germany) and to control the relay of the electromagnet and the stepper motors of the needle valves. The relay circuitry can be referred to in Appendix D.

During the operation of the decanter, the electromagnet is controlled using a relay and Arduino so that the magnet is supplied with power for 5 s every 20 s. The optical sensors approximately determine the position of the phase interface in the decanter. Using the stepper motors controlled by the Arduino, the needle valves can be positioned to inhibit one or the other liquid flow. For example, if the phase interface rises above the upper optical sensor, the upper valve is closed. Consequently, if the pressure in the decanter remains the same, more liquid will flow through the lower outlet. If the phase interface falls below the lower phase interface, the valve at the lower outlet is closed. If the interface is between the two optical sensors, both valves are open.

Since the strongest possible magnetic field is needed to ideally support the sedimentation of the ferrofluid, continuous operation of the magnet can result in a highly viscous fluid in the lower part of the decanter. Due to the magnetic attraction, the ferrofluid then does not flow through the lower connection as intended but collects at the bottom of the decanter. For this reason, the magnet is not operated continuously but is switched on for 5 s every 20 s. On the one hand, this allows the magnetic phase to flow off better and, on the other hand, the electromagnet can be supplied with a higher voltage than permitted for continuous operation. This is because the solenoid (electromagnet) can cool down during the deactivated periods and therefore does not exceed the maximum permissible temperature of about 90 °C even at higher voltages.

If the phase ratios of the flow entering the decanter are unequal, one of the two phases may back up in the decanter due to the lack of a selective membrane. This effect can lead to the light phase being discharged through the lower outlet or the heavy phase through the upper outlet. To prevent this, the phase interface inside the decanter is controlled so that it is always at a distance from both outlets.

**Figure 14:**
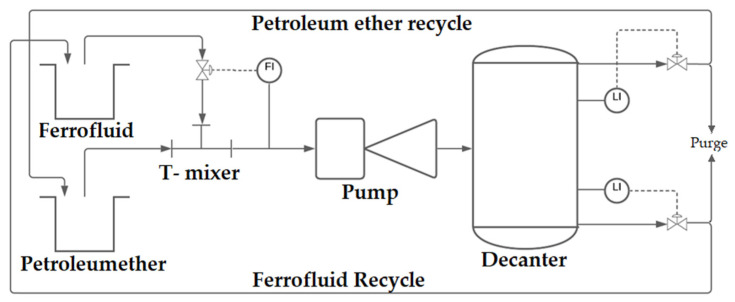
PID flow diagram of the entire process.

The phase boundary surface determined by the optical sensors is decisive for the valve positions of the outlets. It can be shown that using the pulsed magnetisation and the proportional control of the output currents, continuous phase separation can be carried out. Employing the sedimentation of the ferrofluid supported by the electromagnet, the selective membrane can also be dispensed off. Both output streams of the decanter can be fed to the storage tanks of the magnetic gear pump, thus allowing a controlled continuous circulation of both fluids. The structure of all components is shown as a pipe and instrument flow diagram (PID flow diagram) in Figure 14. 

The ferrofluid and the kerosene are drawn into the T-mixer by the vacuum generated by the pump. An optical sensor behind the T-mixer determines the phase ratio and regulates this using the magnets along which the ferrofluid supply line is guided. From the pump, the flow is pumped into the decanter. Using the needle valves, the decanter’s level sensors enable a stable position of the phase interface and thus the leakage of ferrofluid and kerosene through different outlets. Ferrofluid and kerosene can then be returned to the storage tanks. A major advantage of the setup described is its minimally invasive nature. To make a similar setup possible without the concepts described, it would be necessary to intervene in the flow at several points. The phase ratio in the slug generator is controlled using needle valves. The two-phase flow could be conveyed using various pumps which, however, are positioned upstream of the actual slug generator in all cases. Influencing the slug lengths downstream of the generator would not be possible with a classical setup. A selective membrane would usually be used for the continuous separation of the two-phase flow. The controlled valves mean that this membrane and the associated pressure losses can be dispensed with.

## 5. Comparison Studies

The idea of exploiting the interactions between ferrofluids and magnets to promote flow has been addressed in various scientific papers. Four of these concepts are briefly presented below and compared with the pump presented in this paper based on some characteristic values.

In their work, Kurtoğlu et al. considered various setups to promote single-phase and two-phase flow through a microcapillary [21]. The most promising setup consists of two eight-sided rotors with neodymium magnets on the sides. The required magnetic field gradient is generated in this setup by the rotation of the eight-sided cylinders. The two cylinders and the magnets embedded in them are coordinated in such a way that there is always a magnet near the microcapillary. This magnet then attracts the slug that is active at that moment. Now, the magnet turns away, another magnet is brought into position by the second cylinder in such a way that the slug continues to be conveyed through the capillary. 

Hatch et al. have described a pump that uses the ferrofluid phase only as a pump body to pump a magnetically inert phase [26]. This is done by using two neodymium magnets placed around a circular channel with an inlet and outlet. One magnet is stationary and holds only a portion of the ferrofluid at the exit point. By moving the second magnet along the channel, part of the magnetically active phase is drawn through the channel. This creates a vacuum that draws in new fluid through the inlet of the channel. In the further course of the pump cycle, the fluid is pumped out through the outlet. This concept is very similar to that of a classic piston pump. Here, the magnetic slug moving through the channel corresponds to the delivery piston and the entirety of the ferrofluid to the outlet valve [26].

The pump concept presented by Yamahata et al. again has great similarities to a piston pump [28]. The similarity here is even stronger, as classical valves are used for the supply and discharge. A ferrofluid slug in a microchannel is moved by an external magnet in such a way that a pressure differential is generated in one direction of movement and a magnetically inert fluid is sucked in through one valve. In the opposite direction of movement, overpressure is generated, and the magnetically inert fluid is pumped through the second valve. The pump is made from a total of seven polymethyl methacrylate layers. With only one pump, as with a simple piston pump, it is not possible to generate a continuous volume flow. Due to the pump’s motion sequence, the flow conveyed by the pump pulsates strongly [28]. 

In the pump developed by Ashouri et al., a process is presented that functions similarly to a piston pump [30]. However, it should be noted that a special design of the microchannels enables operation without valves. The ferrofluid slug shown in black is set in motion by an external magnet and generates the pressure differences required for pumping. With this pump, in addition to the pulsation known from piston pumps, a backward movement of the fluid can be observed. Due to the valve-free design, fluid is sucked back into the pump from the outlet in step (b). 

Within the scope of this work, a pump is developed which is designed to convey two-phase flows, whose special feature is many magnets, each of which interacts with a ferrofluid slug. The ferrofluid slugs set in motion in this way generate a pressure differential which, on the one hand, sets the magnetically inert phase in motion and, on the other hand, is large enough to convey a continuous flow into the pump. Due to the rotational movement of the disc, the magnets can always convey new slugs without going through a passive phase. The pulsation of the delivery is therefore only dependent on the drive by the stepper motor. Table 3 lists some characteristic data of the pump types considered and compared with the data from the pump developed in this work. All results refer to a continuously operated pumping of a flow in microchannels. Diameter, cross sectional area and channel cross-section describe the type of microchannel in which the flow is conveyed. The maximum counterpressure that can be overcome, and the maximum achievable volume flow are shown as characteristic values. In addition, the number of magnets that simultaneously interact with the ferrofluid and set it in motion is listed. 

It should be noted that different types of ducts are used for the concepts shown. Although the cross-sectional areas and the types of channel cross-sections differ here, they allow a comparison of the different concepts due to similar orders of magnitude. This work differs from the other concepts in the number of magnets that are in contact with the ferrofluid. Only Kurtoğlu [5] also uses several simultaneously moving magnets to set the flow in motion. In the other works, neodymium magnets of a similar size are also used, but only one magnet interacts with the ferrofluid in the microchannels. In these cases, the flow is set in motion by periodic movements. 

For the pressure loss and the volume flow, it can be observed that the pump developed in this work achieves significantly higher values than in the other observations. This is mainly due to the significantly larger number of magnets acting simultaneously with the flow, as with a larger number of magnets, stronger interactions between the magnets and the fluid can be observed which result in higher delivery volume flow and developed back pressures.

## 6. Improving the Developed Magnetic Pump with Automatic Slug Length Variability

The pump described above can be used to continuously deliver a two-phase flow through a microcapillary. Instead of exchangeable gear wheels, as shown in the previous case, a design is made to change the mean slug spacing (for more details on the term, refer to Appendix B) during operation. However, since it has been shown that the conveying speed and the counterpressure also have an influence on the slug length and the distances to each other that are ultimately achieved, it may well be necessary to integrate the slug length distance as a manipulated variable in a controller. Therefore, in the following, a concept is developed which allows that and, thus, also the control of the average slug spacing during operation.

**Figure 15:**
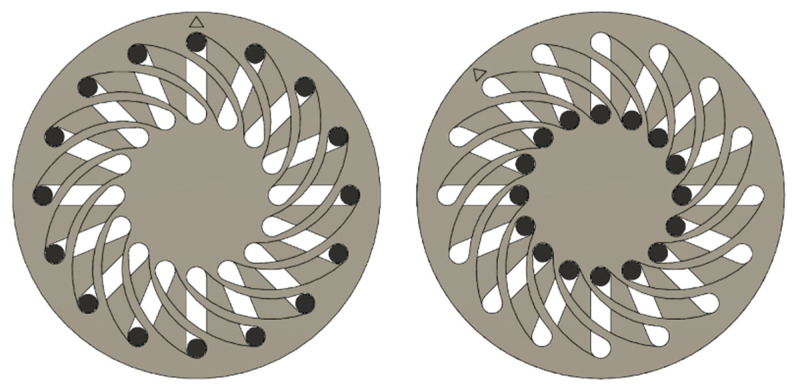
Maximum and minimum diameter of the bolts as a simplified representation of the mechanism. The triangle on the top disk indicates the disk turning 45° to the left.

The basic concept of this revised pump is that the distances of a constant number of objects on a circular path change as the diameter changes. To exploit this effect, i.e., to move physical objects along a circular path of variable diameter, two superimposed discs, capable of rotating independently are needed. Sickle-shaped slots in one disk and straight slots in the other disk result in a constant number of equally large passages through both disks. If bolts or pins are now pushed through these passages, the diameter of the circle describing the centres of all bolts changes when the plates are rotated against each other. This process is illustrated in Figure 15. 

In this case, there are 16 slots in both discs through which 16 bolts are guided. By turning the upper disc 45° to the left, the bolts are pushed into the centre. To transfer this mechanism to the pump described in Section 2 the combination of discs and bolts shown above is needed twice. One mechanism changes the radius and the distance between the magnets, the other changes the diameter of the circular path on which the capillary lies. The entire pump requires a total of four discs, which are assembled with a total of 32 bolts to form two of the mechanisms described above. One to change the diameter of the capillary track and one to change the diameter of the magnetic track.

**Figure 16:**
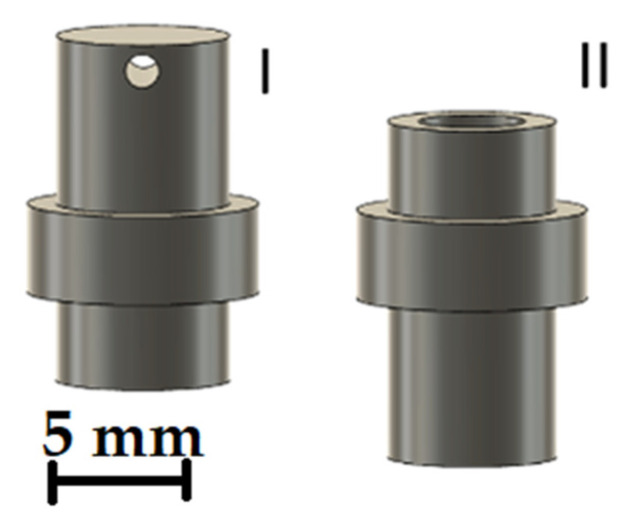
CAD drawing of the bolts for the capillary (**I**) and the magnetic support (**II**).

**Figure 17:**
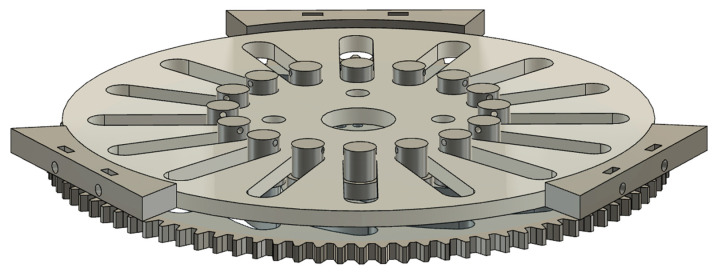
Switching mechanism to change the diameter of the capillary path.

Different bolts must be made for the two mechanisms. Figure 16I shows the bolt for the capillary track. The capillary can be guided through the hole in the upper part of the bolt. The bands in the middle prevent the bolts from slipping through the plates and keep the discs at a constant distance from each other. Figure 16II shows the bolt for the magnets. A round neodymium magnet with a diameter of 6 mm and a thickness of 3 mm is inserted in the upper part. The discs for the capillary mechanism are manufactured in such a way that the lower of the two discs has a gear rim to enable a drive—using a second gear wheel. The upper disc has mountings attached to it that allow a total of three stepper motors to be attached. Both discs and the bolts in the corresponding slots are shown in Figure 17. 

**Figure 18:**
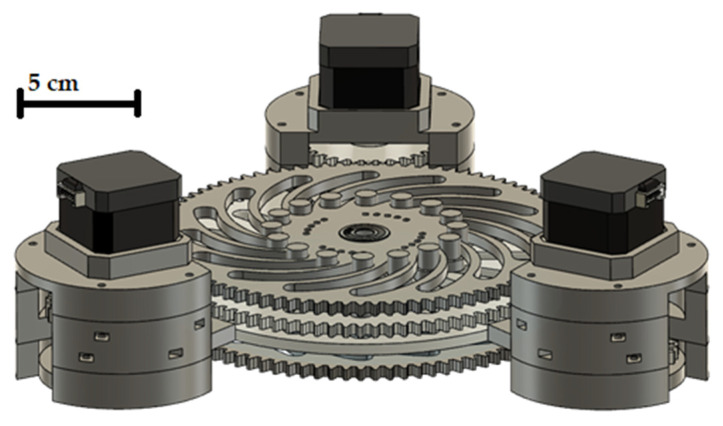
CAD drawing of the entire structure of both mechanisms with stepper motors.

For the magnetic track, the mechanism is made like the one shown in Figure 17. In this case, sprockets are attached to both discs so that both discs can be driven independently by stepper motors. Figure 18 shows the entire construction as a CAD graphic. As can be seen in Figure 18, a total of three holders for stepper motors are attached to the second-lowest disc. A gear wheel is attached to each of the stepper motors, which drives one disc each. The motor holders also serve as supports for the pump. A ball bearing is embedded in the centre of each of the four discs to centre all discs using a screw. In the lower part of the support, there are also ball bearings in which the drive wheels are mounted. 

**Figure 19:**
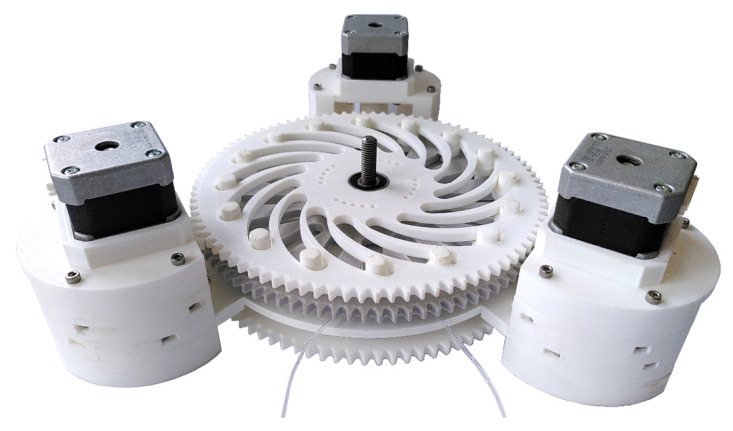
Assembled pump with all gears and motors.

All parts are shown except for the stepper motors, the ball bearings and the screws are made with a 3D printer. A total of 45 parts are printed and installed. The individual parts of the supports and motor mounts are connected using a total of 30 M3 screws and nuts. The stepper motors are controlled using TMC stepper motor drivers, which are controlled by an Arduino. The mechanisms described are manufactured and assembled as shown in Figure 19. 

For the mechanism described above to be used as a pump for ferrofluids, a few things must be considered. The orbit of the embedded magnets must always correspond to the orbit of the capillary to allow interaction between magnets and ferrofluid. Therefore, the switching of the diameters must be done simultaneously. To change the diameter of the capillary or the magnets from maximum to minimum, the two discs must be operated with a phase difference of 45°. For the mechanism of the capillary, this is achieved by driving only the lowest disc. Since the second disc does not move, this changes the position of the bolts.

However, in addition to the rotation for the diameter change, the magnets built into the upper mechanism must be in constant motion to keep the flow in the capillary moving. Therefore, both discs are driven independently of each other. In regular conveying operation, both discs run at the same rotational speed. If the diameter is changed during operation, one of the gears runs slower or faster. In this way, a relative rotation of the discs to each other can be generated, causing the bolts to move inwards or outwards. This relative speed change must occur parallel to the change of the capillary diameter so that the continuous conveying does not break off. This is explained in more detail with an example.

In regular conveying mode, the two discs of the magnetic path rotate at a rotational speed of 2 rpm. If the pins are positioned so that they describe the smallest possible diameter, this results in a flow velocity of 0.5 M·min^−1^ in the capillary. The mean slug spacing is 15.7 mm. Now, the mean distance of the slugs in the capillary is increased. To do this, the magnetic and capillary paths are switched to the largest possible diameter. Switching the diameters is done within 10 s in this example. For the capillary mechanism, only the lowest wheel must turn 45° within 10 s to push the bolts outwards. Therefore, it is driven at a rotational speed of 0.75 rpm for this period. 

Both discs of the magnetic bolts rotate before, during and after the changeover. However, for both discs to rotate 45° to each other, the upper wheel is driven at a rotational speed of 2.75 rpm during this period. After the switching process is completed, the speed is reduced again. By switching both mechanisms at the same time, it can be ensured that magnets and capillaries are on top of each other during the entire process. Due to the larger diameter and thus also circumference, the flow velocity is about 0.94 m/min. The slugs now have an average distance of 29.45 mm under ideal conditions.

**Figure 20:**
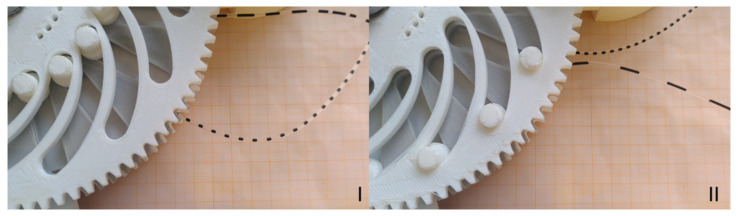
Stationary phase ratios and slug lengths before (**I**) and after (**II**) switching.

The example described is carried out with the manufactured pump. Here, the phase ratio is controlled to 0.5 by the solenoid control. The continuous delivery did not stop during the changeover and a stationary state is reached after a short run-in phase. The stationary states before and after switching are shown as a photo in Figure 20.

**Figure 21:**
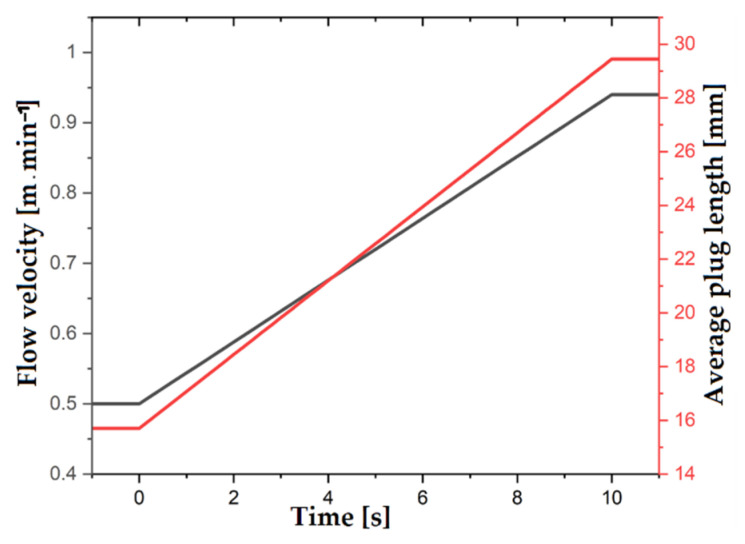
Dynamic change of the target speed and the target slug length throughout the changeover process.

It is to be noted that the capillary bearing has shifted in the switching. In part I of Figure 20, the lower capillary is the suction side and, in part II, the upper capillary is the suction side. This can be seen from the short slugs in the capillary, and such short slugs are produced directly by the T-mixer. During the switching process, the set values of the velocity and the average slug length change due to the changing geometries. The dynamic transition of these values is shown in Figure 21. On the one hand, intermediate values of the mean slug length and the speed can be achieved by interrupting the switching process after an arbitrary time. On the other hand, the dynamic change can be made faster or slower by increasing the speed of one gear or decreasing the speed of the other gear. This would lead to faster or slower changes in speed and average slug length.

It is thus shown that switching during continuous pumping with the described pump is successful. The characteristic values of both operating states are shown in Table 4. As can be seen here, the phase ratios before and after switching the mechanism have minimal deviations from the set values. The average slug distance of both states is also comparable to the value specified by the magnet position. These discrepancies could be due to the larger distances between magnet and capillary in this setup which could lead to the interactions between magnet and ferrofluid being weaker, making the slip described in Section 2 to occur more frequently. However, this slip can be reduced by using stronger magnets and low tolerances in the production of the pumps.

Although the pump described can be switched during operation using the integrated mechanisms and thus produces different average slug lengths, several deficits are nevertheless identified during the commissioning of the pump that requires further revision and improvement. All points are explained using the example explained above. First, the problem of changing velocity is addressed. Since not only the distances between the magnets change due to a further diameter of the magnetic path, but also the speeds of the magnets themselves, a significantly higher flow speed can be observed after switching. If this change in velocity is not desired, it must be compensated for by a steady adjustment of the rotational speed during the switching process. 

Another factor could be the inaccuracies involved in the manufacturing of the components. Due to the additive manufacturing process, rough surfaces and inaccuracies occur on the surfaces compared to classic manufacturing processes such as injection moulding or precision machining. Especially with moving parts, these inaccuracies can lead to the jamming of various parts during continuous operation. Since there are a total of 39 moving parts in the pump described, such malfunctions occur quite frequently, and long-term operation is difficult. 

Another problem is related to the phase ratio control described in Section 3. The strongly fluctuating flow velocities caused by this pump and the correspondingly fluctuating pump pressure are disturbance factors for the control described. However, the control described, which works by changing magnetic fields, is only designed for small changes in pressure and velocities and is therefore not robust enough to correct such large fluctuations. By fundamentally revising the controller structure, this could be adapted and allow the pump to operate. Despite unchanged control, the phase ratio before entering the pump is not constant. With the larger diameter of the circular path, i.e., the higher flow velocity, a higher proportion of ferrofluid can be seen in the capillary. However, the influence of different flow velocities on the phase ratio and the associated control has not yet been investigated in detail. 

## 7. Conclusions

The results achieved within the framework of this work will be briefly summarized along with a brief overview of the potential of the described topic and the direction in which further investigations are to be carried out. Initial reaction technology applications, automation, and considerations for scale-up are discussed. This work started with the investigation of the potential of ferrofluids in microfluidics and the interactions of ferrofluid slugs in two-phase flows with external magnetic fields. 

Then, the interactions of electromagnets are investigated in terms of their potential for the continuous promotion of two-phase flows, following the work of Joung et al. [24] and mathematical simulations carried out to simulate electromagnetic forces. Employing both approaches, rudimentary pumping processes are investigated, but an actual pumping of the flow could not be achieved. The main reason for this is the strong local fluctuations in the magnetic fields, which caused the deformation and rupture of the slugs. By using permanent neodymium magnets, it is possible to achieve a continuous flow. For this purpose, up to 32 magnets are embedded in a gear wheel and guided in the immediate vicinity of the two-phase flow. The slugs set in motion in this way generated a pressure differential that is strong enough, not only to convey the continuous, magnetically inert phase but the pressure differential is also sufficient to continuously generate a two-phase flow using a T-mixer upstream, which could then be conveyed by the pump. A total of three different gearwheels could also be used to create slugs of different lengths. 

A control system is designed to regulate the phase ratio of the flow, which is essential for pumping. First, a strong electromagnet is placed in the immediate vicinity of the ferrofluid supply line. Depending on the phase ratio, the voltage with which the electromagnet is supplied is changed. The viscosity of the fluid changes due to the different magnetic fields and the inflow can thus be regulated. An existing decanter design is upgraded to continuously separate the flow, without a selective membrane. The sedimentation caused by gravity is supported in this decanter by an electromagnet. Due to this enhanced sedimentation and a level control made possible using photoelectric sensors and needle valves, the continuous phase separation can also be carried out without the selective membranes usually used.

To further investigate the potential of the pump described, it is updated in such a way that it allows the slug lengths to be changed during operation using a mechanism. Using mounted neodymium magnets and a mounted diaphragm, the orbital radii and, thus, the magnet distances can be changed during operation. To classify the results achieved, the developed pump is compared with four similar concepts from the literature based on the maximum achievable pump pressure and the maximum delivery volume flow. This comparison showed that the pump described in this work can both deliver the largest volume flow and overcome the largest pressure difference.

To make the described overall concept interesting for technical reaction investigations, it is first necessary to identify a reaction system that does not interact unwantedly with one of the two phases. In particular, the surfactant shell of the magnetite nanoparticles must be considered. Initial laboratory tests have shown, for example, that the surfactant shell of the nanoparticles decomposes under the influence of hydrogen peroxide (an example of a mild oxidant). The then exposed particles tend to coagulate and fall out of the solution. The characteristic of the single domain, which is necessary for the described processes, is thus lost. Under the influence of hydrochloric acid, not only the surfactant shell decomposes, but also the nanoparticles themselves dissolve completely, depending on the acid concentration. Therefore, when selecting a reaction system, it is important to ensure that no reactants react with the surfactant shell or the magnetite.

Another possibility to use the described concept procedurally is continuous extractions. Here, the reactive decomposition of the nanoparticles can be estimated much better. Using the precisely adjustable slug lengths and phase ratios, a detailed statement about the reaction conditions could be made here. Counter-current operation would also be interesting for reaction technology. In this case, the ferrofluid slugs are pumped in one direction by the described pump, while the magnetically inert phase could be pumped in the other flow direction, for example, using syringe pumps. Initial tests have shown that this process can be implemented in principle, but that the continuous supply of ferrofluid represents a major hurdle.

As has been shown in the course of this work, it is possible to influence both the phase ratio and the average slug spacing by exploiting the magnetic activity of the ferrofluid. However, it has not yet been possible to demonstrate a change in both variables simultaneously during operation. However, a revision and ultimately a precision mechanical manufacturing of the pump can be used to achieve this. Using robust control loops, it would then be possible not only to change both variables during operation but also to stabilise them in terms of control technology.

**Figure 22:**
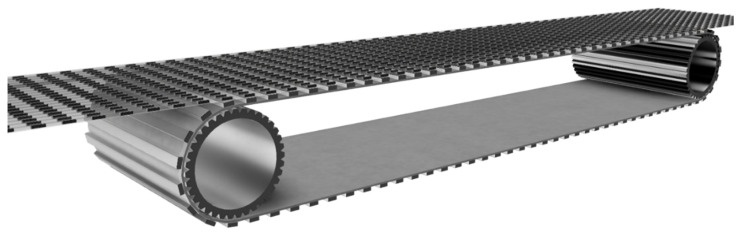
CAD drawing of a theoretical parallelisation using a conveyor belt.

Since process engineering in microchannels is influenced by completely different effects than in larger plants, the scale-up of micro process engineering processes is a very special challenge [3,10,11]. Therefore, scale-up is often achieved by numbering up rather than by moving the processes into larger pipes or vessels. To carry out the pumping and the changing of phase ratios and slug distances simultaneously in several capillaries, a change of equipment is necessary. As shown in Figure 22, several capillaries could be moved simultaneously at the same speed over a conveyor belt. 

Using an elastic conveyor belt and mounted conveyor rollers, the distance between the slugs could even be changed during operation. However, how controlled phase generation and separation is carried out, in this case, remains to be determined. As Kurtoğlu [5] has shown, conveying a two-phase flow using a conveyor belt is possible in principle. Through additional apparatus modification, it is therefore conceivable that parallelisation, as shown, is possible. Reactions involving the oxidation of Methyl oleate, as demonstrated by Gladius et al., clearly benefit from liquid–liquid slug flow operations [3]. Such reactions could be parallelized and made more cost-efficient with such a setup shown below. 

However, whether and how parallelisation is to be carried out depends on many other factors. First, the concepts explained in this paper must be further investigated and operating conditions must be developed through detailed test series. The methods are shown to promote and manipulate the flows certainly show potential for further results and possibilities in the field of microfluidics.

## Figures and Tables

**Table 1:** Maximum capillary length for three operating conditions.

Rotational Speed [rpm]	1.25	2.5	3.75
Maximum Pressure [kPa]	3330	2830	530
Volume flow [µL s^−1^]	0.0078	0.012	0.021
Max capillary length [m]	10.47	5.78	0.61

**Table 2:** Maximum flow rates for the three gears.

No. of Magnets	Volume Flow [µL s^−1^]	Rotational Speed [rpm]
16	13	2.52
24	32	4.98
32	100	11.28

**Table 3:** Comparison between different pumping concepts developed.

	Kurtoğlu [21]	Hatch [26]	Yamahata [28]	Ashouri [30]	This Work
Diameter [µm]	254	1000	1000	N/A	1000
Cross-section [mm^2^]	0.051	0.785	1	0.292	0.785
Channel type [-]	Round	Round	Quadratic	Rectangular	Round
max ΔP [kPa]	N/A	1.2	2.5	0.75	3.3
max $\dot{V}$ [µL s^−1^]	0.0123	0.763	0.5166	11.6	100
No. of magnets [-]	16	1	1	1	32
Flow type [-]	Two phase	Single phase	Single phase	Single phase	Two phase

**Table 4:** Characteristic sizes of the modified pump.

State	I	II
Rotational speed [rpm]	2	2
Flow velocity [m·min^−1^]	0.5	0.9
Target average slug spacing [mm]	15.7	29.4
Achieved average slug spacing [mm]	14.6	26.9
Target phase ratio [-]	0.5	0.5
Achieved phase ratio [-]	0.43	0.60

**Figure A1:**
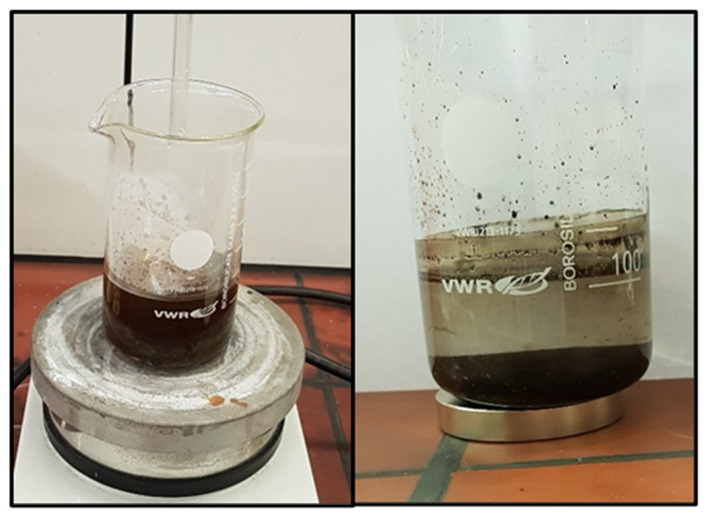
Produced magnetite particles from the bottom-up process (**left**). The same vessel on a strong neodymium magnet (**right**).

**Figure A2:**
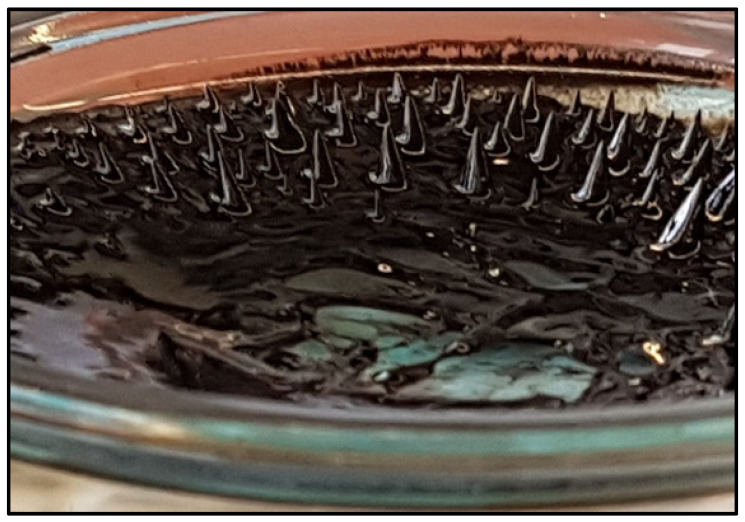
Observed Rosensweigin stability in the prepared Ferrofluid.

**Figure A3:**
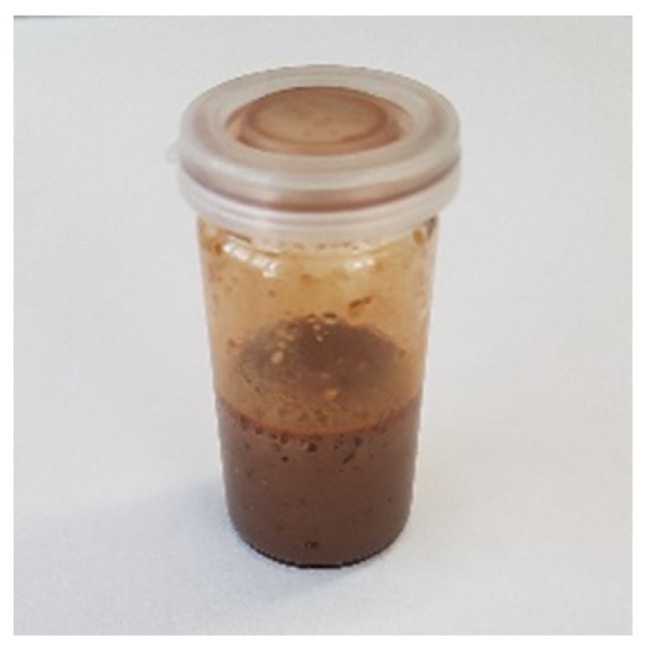
Alternative ferrofluid coated with oleic acid, polyethylene glycol and agar a few hours after preparation.

**Figure A4:**
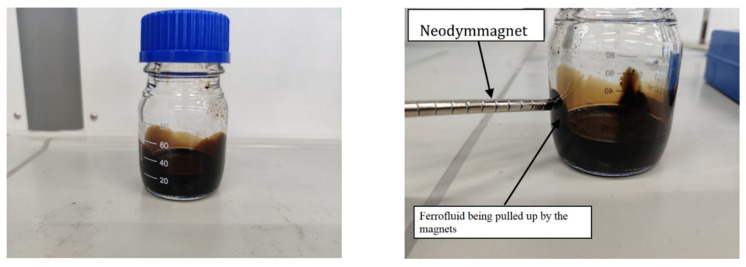
Aqueous ferrofluid (tetramethylammonium hydroxide) after preparation (**left**). The reaction of the same ferrofluid after 3 weeks of standing under the influence of a neodymium magnet (**right**).

**Figure A5:**
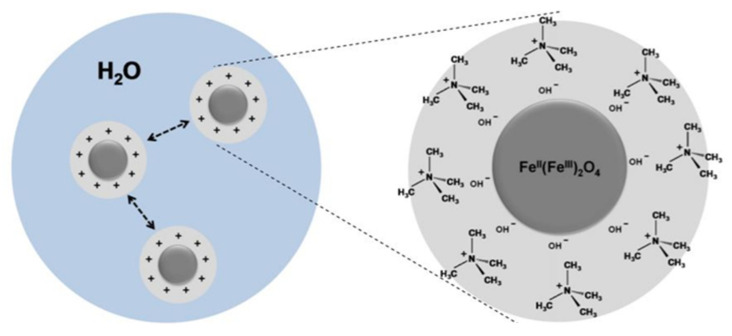
Schematic representation of magnetite particles suspended in water with a positively charged nanosphere of tetramethylammonium cations.

**Figure A6:**
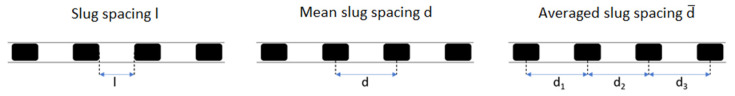
Difference between slug spacing, mean slug spacing and averaged slug spacing.

**Figure A7:**
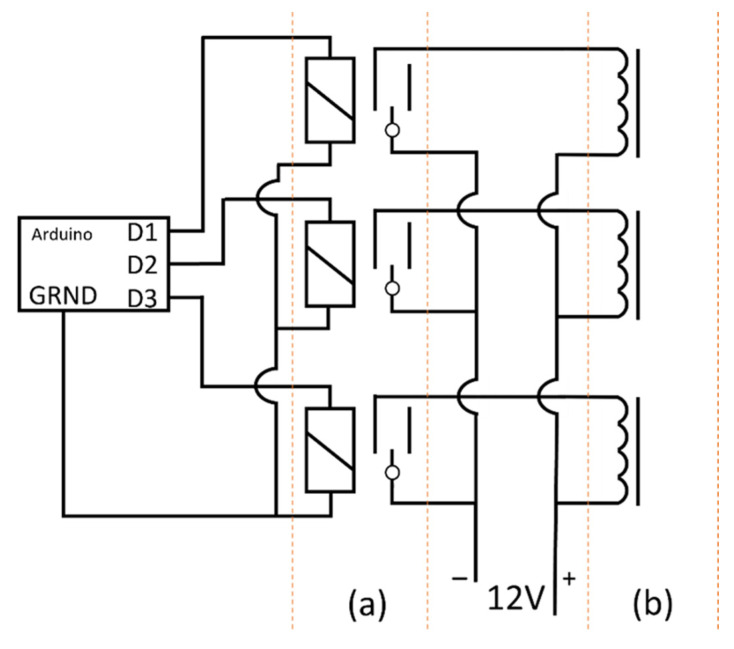
Circuit diagram of the described setup consisting of an Arduino, three relays (**a**) and three electromagnets (**b**).

## Appendix A. Preparation of Ferrofluid

Ferrofluids describe a colloidal system with magnetofluid properties. The combination of both phenomena is not found in nature. The two-phase suspension consists of the dispersed nanoparticles and the carrier medium with hydrophobic or hydrophilic character [33].

A distinction is made between a bottom-up and a top-down process. Accordingly, the nanoparticles emerge from a chemical reaction (bottom-up) or are ground from larger units to the required size (top-down). The top-down process requires a grinder and a mechanical transmission of force to grind larger magnetite particles. The equipment and energy required, and energy costs are disproportionate to those of the chemical bottom-up method, in which the particles can already be obtained in the nanoscale range as the product of a reaction [34,35]. The underlying overall reaction provides for hydroxide ions to be added to the iron ions of different oxidation states. Oxidation states are provided with hydroxide ions, to subsequently form the ferrosoferric oxide with the chemical designation iron (II, III) oxide [36]:

Fe^2+^ + 2Fe^3+^ + 8OH^−^ → Fe_3_O_4_ + 4H_2_O

In the following, Fe_3_O_4_ is called magnetite. The way the hydroxide ions are supplied has a decisive influence on the spectrum of the particle size distribution. There is a tendency to expect larger particles when hydroxide ions are added quickly. The dosage rate of the hydroxide ions and their concentration should be kept low for a narrow particle size distribution with small diameters [36].

According to the chemical reaction, Fe^2+^ and Fe^3+^ must be combined in a molar ratio of 1:2 with eight times the hydroxide ions. Fe^2+^ and Fe^3+^ originate from acidic solutions with FeCl_2_ and FeCl_3_, while the hydroxide ions originate from an ammonium hydroxide solution. For better handling, it is advisable to prepare only smaller amounts of magnetite and repeat the step if necessary.

With the aid of a strong neodymium magnet, the magnetite particles are held on the bottom and the clear supernatant can be trivially decanted. Figure A1 shows the settling of the particles in the magnetic field. In the pure gravitational field, the settling time of the particles is several hours, whereas with a magnet it is only seconds. The magnetite particles of a nanoscale dimension are preserved. Due to the high surface area, the particles reflect light in such a way that they appear black. Further magnetite can be produced for higher concentrations or larger quantities.

Once the desired quantity of manganese titanium particles has been produced, they can be subjected to the coating process. The generation of nanoparticles with ferromagnetic properties, such as from magnetite, does not pose the greatest challenge in the preparation of the ferrofluid in a carrier medium. The produced nanoparticles of magnetite must satisfy a macroscopic homogeneity for use in ferrofluid and practical applications. The suspension must be stabilized against sedimentation-induced segregation in the gravitational field and agglomeration in the magnetic field gradient using a multi-stage coating process. Furthermore, it is important to increase the resistance of the magnetic properties against chemical influences and to preserve them over a long time. The oxidation of magnetite from Fe_3_O_4_ to Fe_3_O_3_ is accompanied by a drastic decrease in magnetic properties [37].

For application in a ferrofluid, the magnetite particles must be stabilised against magnetic and gravimetric agglomeration and sedimentation. A glass rod, a shallow dish and a 25% solution of tetramethylammonium hydroxide are needed in the further process. A spray bottle is used to wash up the magnetite particles remaining adhesive in the beaker and the suspension is transferred to a shallow dish. The water used for washing out must be decanted again magnetically to retain the residual moist magnetite particles on the glass dish. In the next step, 2 mL of tetramethylammonium hydroxide is added and mixed by hand with a glass rod until each particle is in contact with the detergent. This suspension is again magnetically decanted and the coated magnetite particles remain. Once the dark supernatant is poured off, the Rosensweigin stability [38] observed as in Figure A2.

The spiked confirmation serves as a quality criterion for a successful production. The ferrofluid is in equilibrium between magnetic force, gravitation, and the surface tension of the carrier medium. The suspension produced meets the definition of a colloidal system and satisfies the superparamagnetic limit. Thus, a ferrofluid is produced in an aqueous carrier medium that can be strongly magnetised and does not exhibit remanent magnetisation. The latter is confirmed by the collapse of the conformation outside a magnetic field. The physicochemical stability of the ferrofluids must first be examined, which corresponds to the quality check of the coating process. The ferrofluid is to be examined concerning the absence of oxidation. For long-term stability, the ferrofluid produced is stored in a sealed container with an air cushion above it for several weeks. Furthermore, it can be assumed that atmospheric oxygen is dissolved in the carrier medium due to the constant stirring during production and, thus, real application conditions are given. An intermediate degassing of the medium from dissolved oxygen does not take place. From the point of view of chemical stability, the ferrofluid must be evaluated about a colour change from black to brown, which is accompanied by oxidation of the magnetite from Fe_3_O_4_ to Fe_3_O_3_ and means a decrease of the magnetic properties. Figure A3 shows an oxidised ferrofluid coated with the detergents, oleic acid, polyethene glycol and agar. A few hours after preparation, a brown colour change can be seen, indicating the oxidation of magnetite. The magnetite particles coated with tetramethylammonium hydroxide show no colour change visible after 3 weeks in the sealed container. A decrease in the magnetic properties is checked by a neodymium magnet, whereupon no decreased reaction of the ferrofluid can be detected (cf. Figure A4). As before, this is a subjective assessment, the result of which can only be verified beyond doubt by measuring the magnetisation curve. This form of magnetic analysis does not take place here.

Sedimentation and agglomeration or their prevention are achieved by the size of the particles and the coating process. Both the magnetic and the gravitational fields set limits on the minimum particle size. The coating process increases the steric demand of a particle, which, on the one hand, increases the buoyancy force and, on the other hand, increases the distance between the particles. The latter ensures that the attractive forces resulting from dipole–dipole interactions and van der Waals forces are overcome. Provided that the interaction potential is positive, repulsive behaviour can be assumed overall. According to Figure A5, hydroxide ions bind to the particle surface, while the tetramethylammonium cation forms a positively charged nanosphere towards the outside, thanks to which additional repulsion occurs.

## Appendix B. Definition of Mean Slug Spacing

For the evaluation and description of the two-phase flow, a uniform description of the effects to be observed is introduced. In two-phase flows, the mean slug distance is an important quantity. In the following, however, the mean slug distance is not used to describe the averaged distance of several slugs, but the distance of the centres of the slugs. If we are talking about the mean value of several distances, this is referred to as the averaged slug distance. The difference between slug spacing, mean slug spacing and averaged slug spacing is shown in Figure A6.

## Appendix C. Pump Performance Curves

When characterising pumps, the representation of the delivery heads as characteristic curves is often used. With the so-called pump curves or throttle curves, the maximum achievable pressures at different volume flows can be described. Using the pump characteristic curve of different pump models, the correct model can be selected for the application in question. If the pressure behaviour of the system is known, i.e., through a system characteristic curve, the pump characteristic curve can be used to determine the operating point for the system. A pump curve can be modelled theoretically but should be determined experimentally for greater accuracy. It should be noted that pump curves are not only dependent on the pump model, but also on the fluid being pumped [39].

A pump curve can also be used to make a statement about the basic technical functioning of the pump. A basic distinction is made here between centrifugal pumps and piston pumps. As seen in Figure 6, the characteristic curve of the piston pump is almost perpendicular to the X-axis with a slight leftward bend for higher delivery heads. With centrifugal pumps, the achievable pressure is a function of the flow rate [40].

Since high-pressure losses occur in microfluidics, the maximum delivery length of a pump in a specific capillary or microchannel is of interest when characterising pumps. To determine the maximum delivery length, the Darcy equation (Equation (A1)) is used for pressure losses in pipelines [41].

(A1) $$\Delta P=\frac{\lambda *L*\rho *v^{2}}{d*2}$$

(A2) $$\lambda =\frac{Re}{64}$$

(A3) $$Re=\frac{\rho *v*d}{\eta }$$

The pressure loss is composed of the coefficient of friction *λ*, the length of the pipe or conduit *L*, the density of the fluid *ρ*, the velocity of the flow *v* and the characteristic length *d*. In the case of laminar flow, the coefficient of friction *λ* can be calculated using Equation (A2) [41]. The Reynolds number in turn is determined by Equation (A3) from the density *ρ*, the velocity *v*, the characteristic length *d* and the dynamic viscosity *η*. The Reynolds number is used, on the one hand, for the pipe friction coefficient in Equation (A2) and, on the other hand, to determine the laminar character of the flow [41].

## Appendix D. Relay Control Circuit Diagram

The circuit diagram for this setup is shown in Figure A7. The relays are each controlled by one of the digital output pins (D1–D3). Each relay then completes a 12-V circuit that activates the solenoids (electromagnets).
